# Cellular investigations with human antibodies associated with the anti-IgLON5 syndrome

**DOI:** 10.1186/s12974-016-0689-1

**Published:** 2016-09-01

**Authors:** Lidia Sabater, Jesús Planagumà, Josep Dalmau, Francesc Graus

**Affiliations:** 1 Neuroimmunology Program, Institut d’Investigacions Biomèdiques August Pi i Sunyer (IDIBAPS), Barcelona, Spain; 2 Service of Neurology, Hospital Clínic, University of Barcelona, Barcelona, Spain; 3 Institució Catalana de Recerca i Estudis Avançats (ICREA), Barcelona, Spain; 4 Department of Neurology, University of Pennsylvania, Philadelphia, USA

**Keywords:** Autoantibodies, IgLON5, Tauopathy, Immunoglobulin subclasses

## Abstract

**Background:**

Antibodies against IgLON5, a neuronal adhesion protein of unknown function, are markers of a novel neurological disorder termed anti-IgLON5 syndrome. The disorder shows a remarkable association with the HLA-DQB1*0501 and HLA-DRB1*1001 alleles, and postmortem studies demonstrate a novel neuronal tauopathy predominantly involving the hypothalamus and tegmentum of the brainstem. The role of IgLON5 antibodies in the pathogenesis of the disease is currently unknown. Here, we have determined the target epitopes of IgLON5 antibodies, the effects of the IgLON5 antibodies in rat hippocampal neurons, and the IgG subclass responsible for these effects.

**Methods:**

HEK293 cells expressing several deletion constructs of IgLON5 were used to determine the epitopes recognized by the serum of 15 patients with anti-IgLON5 syndrome. The role of glycosylation in immunogenicity was tested with PNGase F treatment of transfected cells. Dissociated hippocampal neuronal cultures were used to test by immunocytochemistry the effects of total IgG, IgG1, and IgG4 subclasses of IgLON5 antibodies.

**Results:**

Patients’ antibodies reacted with the immunoglobulin-like domain 2 of IgLON5. Glycosylation was not required for immunoreactivity. The predominant subclass of IgLON5 antibodies was IgG4 but all patients also had IgG1. The mean percentage of specific IgLON5 IgG4 and IgG1 of the samples analyzed by flow cytometry was 64 and 33 %, respectively. In cultures of hippocampal neurons, patients’ antibodies caused a decrease of cell surface IgLON5 clusters that was not reversed after IgLON5 antibodies were removed from the media. The decrease of surface IgLON5 clusters correlated with the rate of antibody internalization. These effects were observed with purified IgG1 but not with the IgG4 antibodies.

**Conclusions:**

IgLON5 antibodies recognize the immunoglobulin-like domain 2 of the antigen, and the reactivity is not dependent on glycosylation. The effects observed on hippocampal neuronal cultures indicate an irreversible antibody-mediated internalization of surface IgLON5. These effects were mediated by specific IgLON5 IgG1 antibodies and suggest a pathogenic role of these antibodies in the disease.

**Electronic supplementary material:**

The online version of this article (doi:10.1186/s12974-016-0689-1) contains supplementary material, which is available to authorized users.

## Background

The recent identification of antibodies against IgLON5, a neuronal cell adhesion protein of unknown function, in serum and CSF of eight patients with similar symptoms was critical to discover a novel neurological disorder characterized by chronic, less often subacute development of gait instability, chorea, dysarthria, dysphagia, episodic central hypoventilation, stridor, and a previously unrecognized sleep disorder with non REM (rapid eye movement) and REM parasomnias and obstructive sleep apnea. Patients frequently develop dysautonomia and acute respiratory distress requiring tracheostomy and intensive care support, likely responsible for the sudden death of some patients [1].

Since the initial description of the anti-IgLON5 syndrome, 17 patients have been identified ([2–4], unpublished cases). All tested patients (12/12) had the same HLA-DQB1*0501 and HLA-DRB1*1001 alleles indicating a genetic susceptibility for this disorder [1]. Although these findings support an underlying immune pathogenesis, patients rarely improve with immunotherapy. Moreover, the postmortem study in three patients demonstrated a novel tauopathy characterized by deposits of hyperphosphorylated tau restricted to neurons and predominantly involving the hypothalamus and tegmentum of the brainstem without evidence of inflammation [1].

It is currently unclear whether IgLON5 antibodies are primarily involved in the pathogenesis of the disorder or represent a secondary (albeit highly specific) effect of the disease. An important step to support the pathogenic role of the antibodies is to show they have direct effects on the target antigen, IgLON5, in cultured neurons. In the current study, we determined the target epitopes of IgLON5, the effects of the antibodies on primary cultures of rat hippocampal neurons, and the antibody IgG subclass responsible for these effects.

## Methods

### Samples from patients with IgLON5 antibodies and controls

Serum and CSF samples were obtained from 15 patients with IgLON5 antibodies confirmed by immunohistochemistry with rat brain and cell-based assay (CBA) [1]. Control sera were obtained from healthy blood donors. Normal human cerebellum for immunoblot studies was obtained from the Neurological Tissue Bank at the Institut d’Investigacions Biomèdiques August Pi i Sunyer (IDIBAPS), Barcelona, Spain. Written informed consent for the storage and research use of serum samples was obtained from patients or representative family members. The study was approved by the ethics committee of Hospital Clínic, Barcelona, Spain.

### Preparation of purified total IgG and IgG subclasses

Total IgG from sera of patients with anti-IgLON5 syndrome and normal subjects were purified with columns of protein A/G sepharose beads (Thermo Fisher Scientific, Waltham, MA, USA). Briefly, 1 mL of serum was incubated with 0.5 mL of protein A/G sepharose beads for 30 min, eluted with 100 mM glycine, pH 2.5, and dialyzed against phosphate buffered saline (PBS). To obtain purified IgG1 and IgG4 fractions, 1 mL of serum was affinity purified with IgG1 affinity matrix (Capture Select IgG1, Thermo Fisher Scientific) packed in a column. The flow-through was kept as a source of IgG4 antibodies. After extensive washing, the IgG1 antibodies were eluted with 0.1 M glycine, pH 2.5, and dialyzed against PBS overnight. The flow-through of the first purification (containing IgG4 antibodies) was incubated with IgG4 affinity matrix (Capture Select IgG4, Thermo Fisher Scientific) and processed as done with the IgG1 fraction. Aliquots were stored at −80 °C at 50 mg/mL.

The protein content of the total IgG and the purified IgG1 and IgG4 subclasses fractions were quantified by Bradford assay (Bio-Rad, Hercules, CA, USA). To confirm the specificity of purified IgG fractions, equal amounts of each fraction were electrophoretically separated in a NuPAGE 4–12 % Bis-Tris gel (Thermo Fisher Scientific) and transferred to a polyvinylidene difluoride (PVDF) membrane (Bio-Rad). Strips were incubated with biotinylated mouse anti-human IgG (Vector Labs, Burlingame, CA, USA), IgG1, or IgG4 (Sigma, St. Louis, MO, USA) and developed by a standard avidin-biotin immunoperoxidase technique. The specificity of the purified IgG1 and IgG4 subclasses was also assessed by immunofluorescence on HEK293 cells transfected with IgLON5 and cultures of rat hippocampal neurons (Additional file 1: Figure S1).

### CBA and identification of the immunodominant region

A human IgLON5 clone from Origene (catalog number SC317071; accession number NM_001101372.1, Origene, Rockville, MD, USA) was used as template to generate commercially (GenScript USA, Inc, Piscataway, NJ, USA) mutated clones with different combinations of the three Ig-like domains. All clones contained the signal peptide (1–30 amino acids (aa)) that targets the protein to the endoplasmic reticulum, the immunoglobulin-like domain 3 (218–306 aa) that contains the epitope recognized by the commercial antibody (rabbit, ab122763 Abcam, Cambridge, UK) (218–289 aa) used to control the efficiency of transfection, and the amino acid sequence containing the omega site (314 aa) that signals the glycosylphosphatidylinositol (GPI)-attachment point (predicted by the software program big PI-predictor IMP, Bioinformatics, Vienna, Austria) [5] (Additional file 1: Figure S2).

The full length IgLON5 clone and the indicated mutants were transfected in HEK293 cells using lipofectamine 2000 (Thermo Fisher Scientific) following standard procedures [1]. After 24 h, transfected HEK293 cells were incubated live with patients’ samples (CSF 1/2 diluted, serum 1/40 or IgG1 and IgG4 subclass fractions 1/50) for 1 h at 37 °C. Cells were fixed with 4 % paraformaldehyde (PFA) for 10 min and sequentially incubated (1/5000) with the IgLON5 commercial antibody (Abcam) for 1 h and goat anti-rabbit Alexa Fluor 594 and goat anti-human IgG Alexa Fluor 488 (Thermo Fisher Scientific) secondary antibodies (1/1000) for 1 h. Results were photographed with AxioCam ICc 3 color camera adapted to an Axioscope Zeiss microscope and analyzed with Zen software (Zen 2012 blue edition 1.1.1.0, Zeiss, Jena, Germany).

### Glycosylation analysis

IgLON5 is highly expressed in the human cerebellum (http://www.proteinatlas.org, Stockholm, Sweden). To study the level of glycosylation of IgLON5, we prepared a human cerebellum protein extract by homogenization in lysis buffer (0.3 M sucrose, 0.5 M EDTA, 1 % TX-100 in presence of protease inhibitors (Sigma)), and 30 μg of the lysate was incubated with endoglycosidases N-Glycosidase F (PNGase F) and O-Glycosidase, which removed N- and O-linked glycans respectively, and neuraminidase, an exoglycosidase that removed terminal sialic acids (New England Biolabs, Ipswich, MA, USA) alone or in combination following the manufacturer’s instructions. Whole protein extracts and deglycosylated extracts were analyzed by immunoblot. The PVDF membrane (Bio-Rad) was incubated with the commercial rabbit anti-human IgLON5 antibody (Abcam) overnight at 4 °C followed by a secondary anti-rabbit-HRP (horseradish peroxidase) antibody (1/1000) for 1 h. Bands were visualized by enhanced chemiluminescence (Amersham ECL western blotting kit, GE Healthcare, Buckinghamshire, UK) in a ImageQuant LAS4000 (GE Healthcare).

To determine if the reactivity of patients’ antibodies was directed to IgLON5 glyco-epitopes, we used two different approaches: (1) An anti-IgLON5-positive serum (1/200) was immunoabsorbed overnight with glycosylated or deglycosylated protein extracts (with PNGase F enzyme) of HEK293 cells expressing IgLON5 or GluR1/GluR2 (AMPA receptor subunits) that were used as negative control. After centrifugation, the absorbed sera were incubated on post-fixed rat brain sections followed by a biotinylated goat anti-human IgG (1/2000, Vector Laboratories) and developed by a standard avidin-biotin immunoperoxidase technique. Results were photographed with an AxioCam MRc color camera adapted to a confocal microscope (Zeiss LSM710) and analyzed with Zen software (Zen 2012 blue edition 1.1.1.0). (2) HEK293 cells were transfected with the full length IgLON5 plasmid as indicated above. Four hours post-transfection, the media was replaced by media containing 2 μg/mL tunicamycin (Sigma) or vehicle as control (DMSO, Sigma). After 24 h with the treatment, cells were fixed with acetone, incubated with an anti-IgLON5-positive serum and developed by indirect immunofluorescence (see above). This study was conducted in fixed cells because deglycosylated IgLON5 protein after tunicamycin treatment is not exported to the cell membrane. Inhibition of N-glycosylation by tunicamycin treatment was confirmed by western blot of transfected HEK293 cell extracts tested with the commercial anti-IgLON5 antibody.

### Analysis of IgG subclasses by CBA and flow cytometry

CBA was performed as above using fluorescent secondary antibodies against the four IgG subclasses (The binding Site, Birmingham, UK). To determine the IgG subclasses by flow cytometry, IgLON5 transfected or non-transfected HEK293 cells were mildly trypsinized and incubated with a mix of IgLON5-positive human serum samples (1/50) and the rabbit-anti-human IgLON5 commercial antibody (Abcam) (1/1000) for 20 min at 4 °C in DMEM (Dulbecco’s modified Eagle medium, Thermo Fisher Scientific) plus 1 % bovine serum albumin (BSA). After washing, cells were fixed with 1 % PFA for 5 min and incubated with the same specific fluorescent secondary antibodies for IgG subclasses used in the CBA (1/500) mixed with a goat anti-rabbit Alexa Fluor 594 (1/1000) for 30 min at 4 °C. Cells were acquired with a BD LSRFortessa flow cytometer and analyzed with DIVA software (Becton Dikinson, NJ, USA). Two-color fluorescent dot plots were applied to calculate the mean fluorescence intensity (MFI) of the binding of the IgG subclasses to the transfected cells. The *X*-axis represents the red fluorescence (transfected cells) and the *Y*-axis the green fluorescence (binding of the serum to the transfected cells) and the quadrant Q2 contains the double positive fluorescent cells (Additional file 1: Figure S3). The MFI measured in Q2 when the serum was incubated with transfected cells was corrected substracting the background MFI obtained with untransfected cells (ΔMFI). The ΔMFI of five different negative controls (normal human serum) obtained in Q2 were used to establish a cut-off threshold using the mean value plus three standard deviations (SD). The sum of the ΔMFI obtained for each sample for the different subclasses was considered as the 100 % binding, and the relative percentage of each IgG subclass was calculated. The values that did not reach the threshold were considered zero for the percentage calculation. Each serum sample was subjected to flow cytometry analysis three times.

### Fab fragments preparation

Fab fragments were prepared as previously described [6]. Briefly, Fab fragments from patient and control IgG were obtained with Pierce Fab preparation kit (Thermo Fisher Scientific). The IgG was digested for 6 h at 37 °C with papain plus cysteine, and the Fab were isolated with a protein A spin column. Concentration was quantified by Bradford assay, and the purity was confirmed by western blot. Neurons were treated with 4 μg/mL of Fab fragments for 3 days. To restore the crosslinking effect, Fab fragments were incubated with anti-Fab secondary antibody 1/250 diluted (Jackson ImmunoResearch Laboratories, West Grove, PA, USA).

### Cell culture, patient antibody treatment, and immunostaining

Primary cell cultures of rat hippocampal neurons were prepared from embryonic P18 day as previously reported [7]. Briefly, dissected hippocampi were trypsinized with 0.25 % trypsin (Thermo Fisher Scientific) for 15 min at 37 °C and mechanically dissociated. Single cell suspension was plated in P35 plates and maintained in Neurobasal medium plus B27 (Thermo Fisher Scientific).

To determine if IgLON5 localized in the synapse, hippocampal neurons were immunostained live with serum containing IgLON5 antibodies. After fixation with 4 % PFA for 5 min, cells were permeabilized with 0.3 % TX-100 and incubated for 1 h with antibodies against excitatory synaptic proteins, including post-synaptic density-95 (PSD95) protein (Thermo Fisher Scientific, 1/200) and synapsin-I (Synaptic Systems, Goettingen, Germany, 1/200) followed by anti-human IgG Alexa Fluor 488 and anti-mouse IgG Alexa Fluor 594 secondary antibodies (1/1000) (Thermo Fisher Scientific).

To determine the effects of patients’ antibodies on neuronal cell surface IgLON5 clusters, 14-day in vitro (DIV14) neurons were incubated with total IgG, IgG1, and IgG4 fractions (at concentrations of 50 μg/mL) from patients and controls for 1, 3, and 7 days; subsequently, neurons were washed, incubated with an anti-IgLON5-positive serum (1/200, used here as a reagent) for 1 h at 37 °C, fixed with 4 % PFA for 5 min, and then incubated with an anti-human IgG Alexa Fluor 488 (1/1000). To determine if the number of synapses was modified by patients’ antibodies, neurons treated for 7 days were immunostained with the indicated antibody synaptic markers (PSD95 and synapsin-I), and the co-localization was quantified using a confocal microscope (Zeiss LSM710) and the Imaris suite 7.6.4 software (Bitplane AG, Zurich, Switzerland).

To identify internalized human IgG, IgG1, or IgG4 along with IgLON5 in neurons treated with patients’ or control samples, the IgG bound to surface IgLON5 was blocked with highly concentrated (1/20) secondary anti-human IgG Alexa Fluor 594 (red fluorescence). Neurons were then fixed with 4 % PFA for 5 min, permeabilized with 1 % TX-100, and labeled with a different (green fluorescence) secondary anti-human IgG Alexa Fluor 488 (1/1000). To confirm that the initial secondary antibody saturated all IgG bound to cell surface IgLON5 preventing the binding of the second secondary antibody, a similar immunohistochemical experiment was conducted in which the neurons were not permeabilized. In this experimental setting, patients’ IgG bound to cell surface IgLON5 was considered completely saturated by the first secondary antibody, if pre-incubation with this antibody completely abrogated the reactivity of the second secondary anti-human IgG.

### Confocal microscopy and image analysis

Twenty immunostained primary dendrites from randomly taken hippocampal neurons after IgG treatments were acquired using a confocal microscope (Zeiss LSM710) with an EC-Plan NEOFLUAR CS, 100/1.3 NA oil objective. The samples were sequentially scanned at 1024 × 1024 lateral resolution and Nyquist optimized z-sampling frequency. For cluster analysis, the images were deconvolved for improving the contrast and resolution with AutoQuantX3 software (Bitplane AG) followed by automatic segmentation using the spot detection algorithm from Imaris suite 7.6.4 (Bitplane). The density of clusters was expressed as number of spots per 50-μm length of dendrite. For three-dimensional co-localization analysis of clusters, a spot co-localization algorithm implemented in Imaris suite 7.6.4 software was applied to determinate the synaptic localization of clusters. All analyses were repeated in three independent experiments.

### Statistics

All the statistic data was calculated by GraphPad Prism 6.0 program. In experiments involving two conditions, the data were analyzed with a two-tailed unpaired Student *T* test. In experiments involving three or more conditions, the data was analyzed using a one-way ANOVA test followed by post hoc analyses applying Bonferroni’s multiple comparison correction. All values are represented as mean + SEM.

## Results

### IgLON5 antibodies target non-glycosylated epitopes in the Ig-like domain 2

**Fig. 1:**
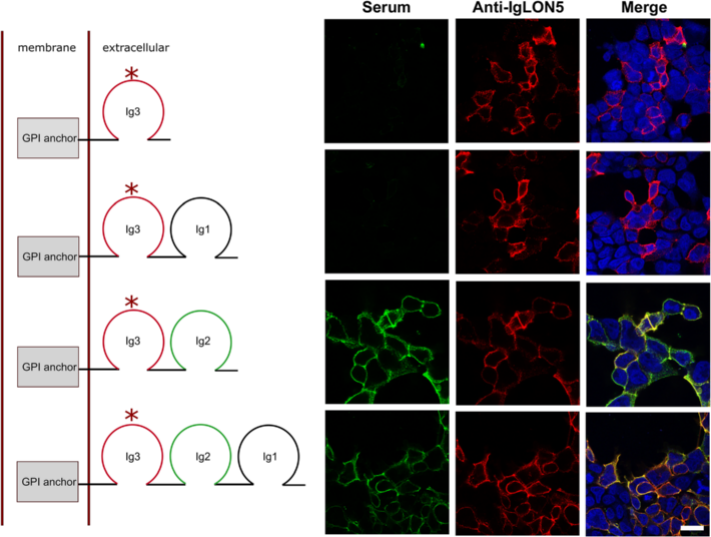
Epitope analysis of IgLON5 antibodies. The diagrams depict the complete IgLON5 (*bottom*) and mutated clones (*left*). Immunofluorescence of transfected HEK293 cells with the full length or the mutant IgLON5 clones (*right*). Rows correspond to the indicated clone in the diagram. The serum with IgLON5 antibodies (*green*) only targets the clones containing the immunoglobulin-like domain 2 (Ig2, *left column*). IgLON5 commercial antibody immunoreactivity is shown in the central column (in *red*) and both reactivities are shown merged in the *right column*. Nuclei counterstained with DAPI (*blue*). Scale bar = 10 μm. The (*) indicates the epitope recognized by the IgLON5 commercial antibody

To determine the immunodominant region recognized by IgLON5 antibodies, HEK293 cells transfected with the indicated mutated clones expressing different combinations of the three immunoglobulin-like domains were tested by CBA using serum samples of 15 patients with the anti-IgLON5 syndrome. All samples reacted with the clone containing the immunoglobulin (Ig)-like domain 2 spanning from 132 to 218 amino acids indicating that the antibodies of all 15 patients recognized the same epitope region (Fig. 1).

The human IgLON5 (A6NGN9 accession number, UniProtKB) has five N-glycosylation sites predicted by Uniprot at amino acids 41, 49, 67, 137, and 288.

**Fig. 2:**
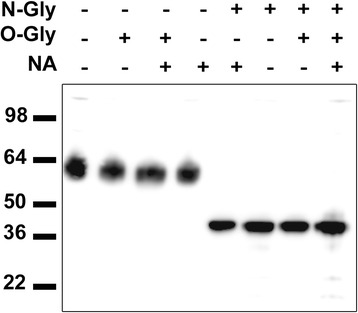
Deglycosylation assay. Western blot of human cerebellum protein extract treated with *N-Gly* (N-glycosidase), *O-Gly* (O-glycosidase), and/or *NA* (neuraminidase) and probed with a commercial rabbit anti-human IgLON5 antibody. The IgLON5 protein is completely deglycosylated only when the N-linked glycans are removed reaching its predicted molecular weight of 36 kDa according to the amino acid sequence

Immunoblots of protein extracts of human cerebellum treated with PNGase F showed a decrease of apparent molecular weight (from 59 to 36 kDa) of the band recognized by the commercial IgLON5 antibody (Fig. 2). The molecular weight of the deglycosylated protein was in agreement with the predicted molecular weight from the amino acid sequence by online tools (http://ca.expasy.org/tools/protparam.html) (Swiss Institute of Bioinformatics). Pre-treatment with O-deglycosylation enzyme or with neuraminidase (see “Methods”) did not change the molecular weight of the detected band indicating that IgLON5 is exclusively N-glycosylated.

**Fig. 3:**
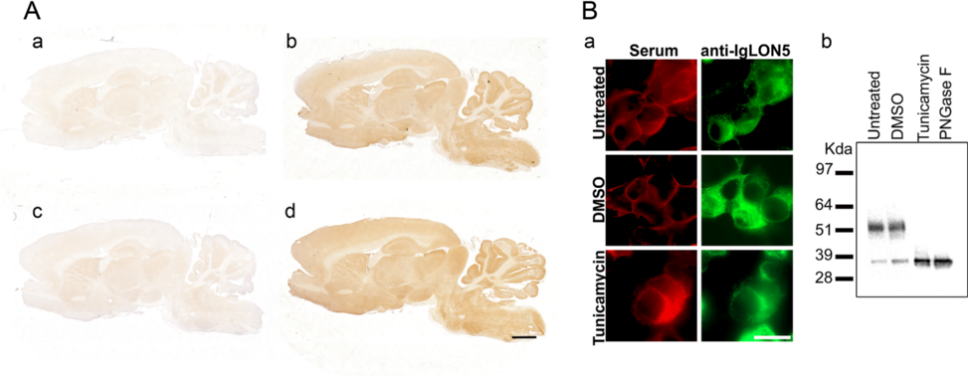
Deglycosylated IgLON5 is recognized by human IgLON5 antibodies. **A** Anti-IgLON5 immunoreactivity in rat brain sections is totally abrogated when the anti-IgLON5-positive serum is preabsorbed with glycosylated (*a*) or deglycosylated protein extracts from transfected HEK293 with IgLON5 (*c*). The immunoreactivity of IgLON5 antibodies is present when the serum is preabsorbed with an unrelated antigen glycosylated (*b*) or deglycosylated (*d*). Scale bar = 1000 μm. **B** *a* Immunofluorescence of HEK293 cells transfected with IgLON5. Prevention of N-glycosylation with tunicamycin treatment does not affect the reactivity of patients’ antibodies with IgLON5. Scale bar = 10 μm. *b* Western blot of extracts of the indicated IgLON5-HEK293 cells shown in (*a*), demonstrating a shift of the molecular weight (from ~52 to 36 kDa) consistent with the deglycosylation of the protein. The findings confirm that the reactivity of patients’ serum antibodies with tunicamycin-treated cells of **A** was directed against deglycosylated epitopes

To analyze if patients’ antibodies recognize a glycosylated epitope, a positive serum was absorbed with a lysate of HEK293 cells expressing IgLON5 or an unrelated protein (GluR1/GluR2) that had been pre-treated or not with PNGase F and the reactivity of the absorbed serum was examined by immunohistochemistry of rat brain. The immunoreactivity of the serum was completely abrogated after absorption with either the deglycosylated or the non-deglycosylated protein extract containing IgLON5 (Fig. 3a) indicating that patients’ antibodies recognize non-glycosylated epitopes.

Furthermore, the treatment of IgLON5 HEK293 transfected cells with tunicamycin (which prevents N-glycosylation) did not change the reactivity of patients’ antibodies with IgLON5. N-glycosylation inhibition by tunicamycin was confirmed by western blot of treated protein extracts (Fig. 3b).

### IgLON5 antibodies are predominantly of the IgG4 subclass

**Fig. 4:**
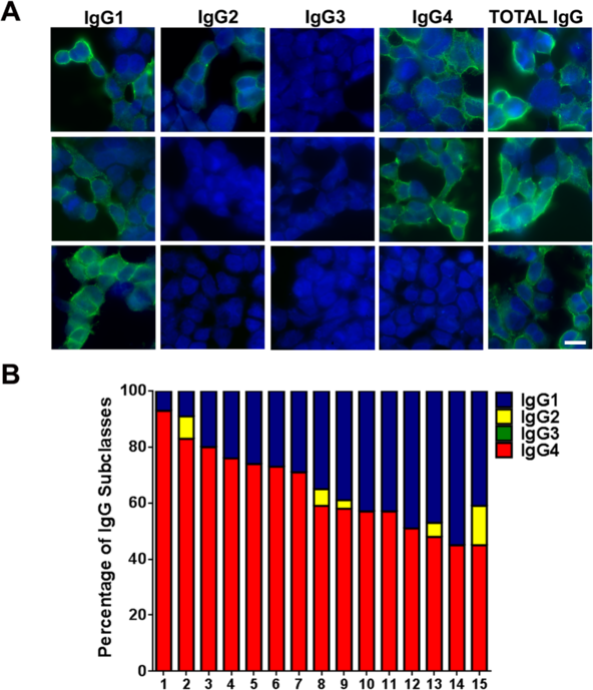
Analysis of IgLON5 antibody subclasses. **a** Example of anti-IgLON5 IgG subclasses in three different sera. HEK293 cells were transfected with IgLON5 and incubated with three sera with IgLON5 antibodies followed by antibodies against total IgG or specific IgG subclasses. Positive immunoreactivity is shown in *green*. Nuclei counterstained with DAPI (*blue*). Scale bar = 10 μm. **b** IgG subclass percentages of IgLON5 antibodies in the 15 positive serum samples analyzed by flow cytometry

Using CBA, all 15 sera had IgG4 and IgG1 antibodies. Four samples also had IgG2 but none had IgG3 antibodies (Fig. 4). The IgG subclass distribution was also confirmed by incubating the serum of the patients on live neurons (not shown). Seven available CSF samples were also analyzed by CBA; five of them matched the results of their respective serum and they were positive for IgG1 and IgG4 antibodies, and in the other two, only IgG4 antibodies were detected.

To quantify the IgG subclass levels, we performed flow cytometry analysis (Additional file 1: Figure S3). The majority of the patients (14/15) predominantly had anti-IgLON5 IgG4 antibodies (Fig. 4). The percentage of specific IgG4 over the total anti-IgLON5 antibodies per sample (sum of the ΔMFI obtained for all the subclasses in a sample) ranged from 45 to 93 % (mean 64.6 %, 95 % CI 56.4–72 %) and for specific IgG1 from 7 to 55 % (mean 33 %; 95 % CI 25–40.8 %). Five patients had also IgG2 antibodies ranging from 3 to 14 % (mean 2.2 %; 95 % CI 0.07–4.4 %). All the samples were negative for IgG3.

### Patient antibodies caused an irreversible decrease of the levels of neuronal cell surface IgLON5

**Fig. 5:**
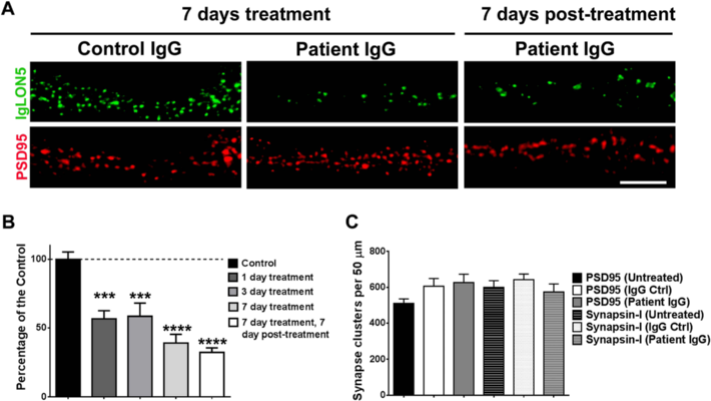
IgLON5 antibodies irreversible decrease IgLON5 clusters on cell surface. **a** Immunofluorescence on hippocampal neurons treated for 7 days (14DIV to 21DIV) with control or patient with anti-IgLON5 IgG. The surface clusters are drastically reduced by the anti-IgLON5 IgG, and the effect is irreversible because 7 days after removing the anti-IgLON5 IgG the reduced number of surface IgLON5 clusters persisted. Scale bar = 5 μm. **b** Quantification of the decrease of IgLON5 clusters on the dendrite surface in a time-course treatment of 1, 3, and 7 days, and 7 days after removing the anti-IgLON5 IgG. ***p < 0.005, ****p < 0.0001. **c** Synaptic markers (PSD95, synapsin-I) were not affected by the anti-IgLON5 IgG treatment

IgLON5 was found widely expressed on the surface of hippocampal neurons without specific localization in the synapse; indeed, in double labeling experiments, only 3.6 % (SD 0.5 %) of IgLON5 clusters co-localized with PSD95 and 4 % with synapsin-I (SD 0.4 %) (Additional file 1: Figure S4). To ascertain the dynamics of antibody effects on neuronal IgLON5, we used 1-, 3-, and 7-day incubations with total IgG from a patient with an average level of IgLON5 IgG1 antibodies (27 % of total IgLON5 IgG antibodies). Patients’ antibodies, but not control IgG, significantly decreased the number of neuronal cell surface IgLON5 clusters in a time-dependent manner (Fig. 5). After 1 day of treatment, only 57 % of IgLON5 clusters remained on the neuronal cell surface. The number of clusters progressively decreased until day 7 (Fig. 5). We next examined whether the levels of neuronal cell surface IgLON5 were restored after removing patients’ antibodies from the media. For this experiment, we removed the antibodies from the media by changing it for fresh media and allowed a 7-day recovery period. After 7 days, the levels of neuronal cell surface IgLON5 did not recover, remaining similar to those obtained after 7-day incubation with patients’ antibodies (*p* = 0.36) (Fig. 5). This is in contrast with the recovery of clusters of NMDAR when IgG from patients with NMDAR antibodies is used (not shown) [6, 8].

Patients’ antibodies did not alter the number of excitatory synapses identified by the number of clusters of PSD95 co-localized with synapsin-I (Fig. 5c).

**Fig. 6:**
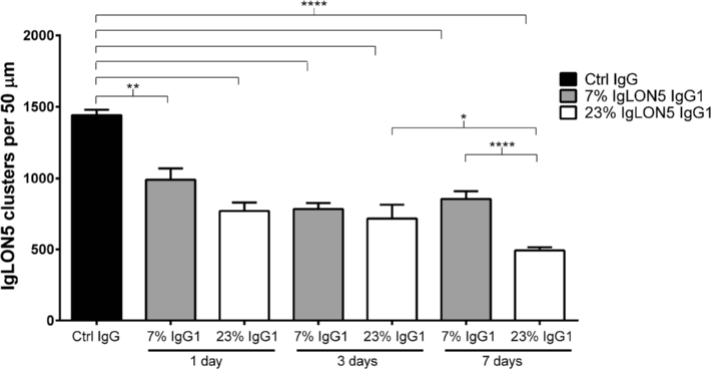
Comparison of the time-dependent decrease of neuronal cell surface IgLON5 clusters after treatment with total IgG of two patients with different levels of anti-IgLON5 IgG1 antibodies. On days 1 and 3, treatment with IgG of the patient with the lower level of IgG1 IgLON5 antibodies (7 % of total IgLON5 antibodies) produced a similar decrease of IgLON5 clusters as the IgG of the patient with average level of IgG1 antibodies (23 % of total IgLON5 antibodies). (**p = 0.001, ****p < 0.0001, comparing with control IgG, one-way ANOVA with Bonferroni’s multiple comparison test). In contrast, on day 7, the sample of the patient with lower levels of IgG1 antibodies did not decrease further the levels of IgLON5, whereas the sample of the patient with average level of IgG1 IgLON5 antibodies produced an additional decrease of IgLON5 clusters (****p < 0.0001) that was statistically significant comparing with day 3 (*p = 0.03)

**Fig. 7:**
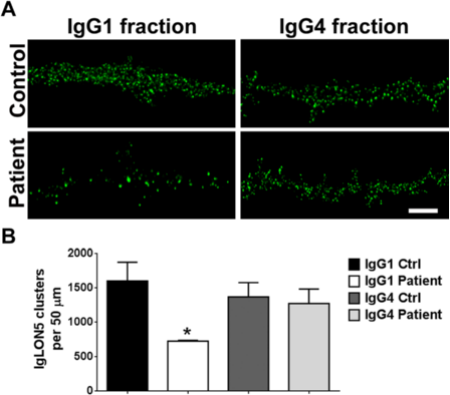
IgLON5 IgG1, but not IgG4 antibodies, cause a reduction of surface IgLON5 clusters. **a** Hippocampal neurons were treated for 3 days with IgG1 or IgG4 fractions from control serum or from a patient with IgLON5 antibodies and immunostained for surface antibody-bound IgLON5. Scale bar = 5 μm. **b** Quantification of IgLON5 clusters. IgLON5 clusters were significantly reduced after treatment with patient IgG1 compared with control IgG1 or patient and control IgG4. *p < 0.05

When the dynamics of antibody effects were determined with a serum in which IgG1 represented only 7 % of the total IgLON5 IgG, a similar decrease of neuronal cell surface IgLON5 clusters was observed at days 1 and 3 but no further decrease was observed at day 7 (Fig. 6). The antibody effects were reproduced using only the IgG1 fraction of patients’ IgG whereas the IgG4 fraction did not change the number of IgLON5 clusters (Fig. 7).

**Fig. 8:**
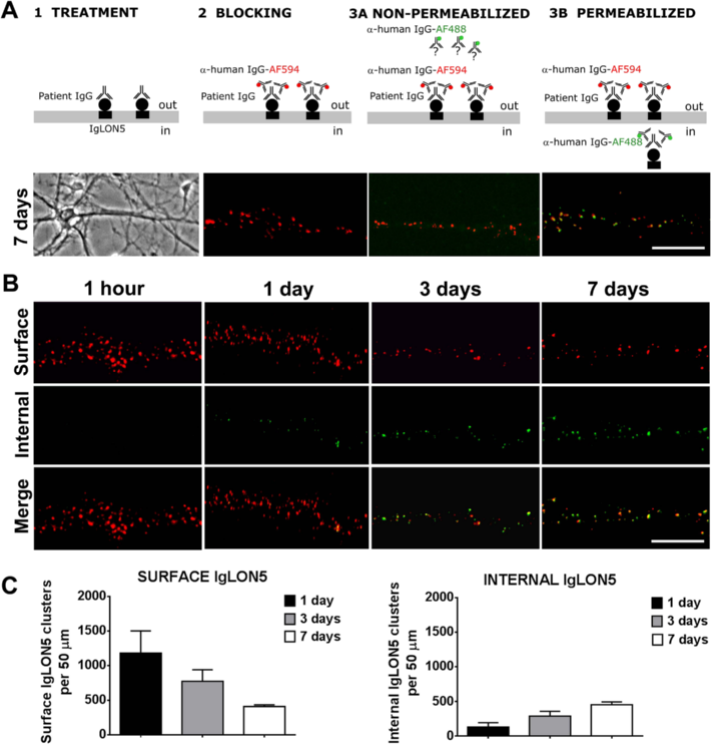
IgLON5 antibodies produce internalization of IgLON5 clusters. **a** Panel 1: Hippocampal neurons treated for 7 days with IgG-positive IgLON5 antibodies. Panel 2: IgG bound to IgLON5 on the dendrite surface labeled live with an excess of anti-human IgG Alexa Fluor 594 (*red*). Panel 3A: The saturation of the neuronal surface prevents that the anti-human IgG Alexa Fluor 488 (*green*) attaches to the surface. Panel 3B: After the neuron is permeabilized and incubated with the anti-human IgG Alexa Fluor 488, the green fluorescence localizes the human IgG attached to internalized IgLON5 clusters. Scale bar = 5 μm. **b** The internalization of IgLON5 clusters is a time-dependent effect and parallels a decrease of IgLON5 clusters on the surface of the dendrite. **c** Quantification of the number of IgLON5 clusters remaining on the cell surface and internalized after the treatment

**Fig. 9:**
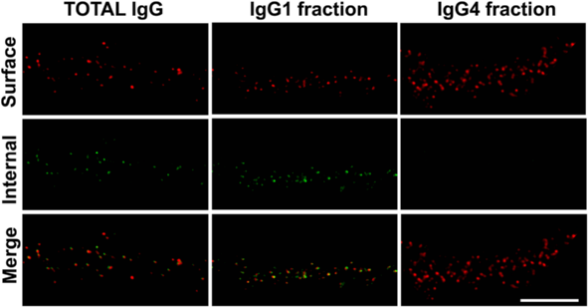
IgG1 IgLON5 antibodies internalize IgLON5 clusters. Hippocampal neurons treated for 3 days with total IgG, IgG1, and IgG4 IgLON5 antibodies. The immunofluorescence strategy to differentiate surface and internal human IgG bound to IgLON5 is conducted as in Fig. 8. The IgG1 antibodies alone could reproduce the same effects seen with the total IgG; meanwhile, the IgG4 did not produce internalization. Scale bar = 5 μm

We next examined whether the reduction of IgLON5 clusters was caused by antibody-mediated IgLON5 internalization. The detection of internalized IgLON5 IgG was noted after 1 day and increased until day 7 in parallel with a decrease of the cell surface IgLON5 clusters (Fig. 8). No detection of internalized IgLON5-IgG occurred if neurons were not permeabilized, indicating that the IgG detected corresponded to internalized antibodies. Similar effects were observed with the purified IgG1 fraction of IgLON5 antibodies, but not with the IgG4 fraction, suggesting that the antibody-mediated internalization of IgLON5 was dependent on the IgG1 antibodies (Fig. 9). No internalized IgG was observed when neurons were treated with control IgG.

Neurons treated for 3 days with Fab fragments obtained from a serum with IgLON5 antibodies had the same density of IgLON5 clusters on the cell surface as neurons treated with control IgG or control Fab fragments (Fab from normal human serum). However, treatment with patients’ Fab fragments plus an anti-Fab secondary antibody (which linked two Fab fragments resembling an intact IgG) produced a significant reduction of neuronal cell surface IgLON5 clusters (Additional file 1: Figure S5). The findings indicate the need of the two arms of the IgG in order to cause internalization of IgLON5.

## Discussion

The findings of this study demonstrate that the antibodies of patients with the anti-IgLON5 syndrome are potentially pathogenic. In addition, we provide the main epitope region of IgLON5, and the subclass of IgG involved in the antibody-mediated internalization of neuronal cell surface IgLON5. The observation that these antibody effects were irreversible is novel and may explain the refractoriness to patients’ symptoms to immunotherapy, suggesting that a prompt diagnosis and treatment may alter the outcome of this often lethal disease.

IgLON5 is the latest identified member of the IgLON family, a subgroup of the immunoglobulin superfamily of cell adhesion molecules [9]. IgLON proteins are highly glycosylated, contain three extracellular immunoglobulin-like domains, and are attached to the lipid rafts of the cytoplasmic membrane through a GPI anchor [10]. Although the physiological role of IgLON5 is unknown, other members of the IgLON family are involved in neuronal pathfinding and synaptic formation during brain development [11, 12] and have been implicated in the pathogenesis of autism spectrum disorders and epigenetics of tumor progression [13, 14].

The identification of the main immunodominant region recognized by IgLON5 antibodies is the first step for a better understanding on how antibodies can interfere with the normal interaction of the antigen with other extracellular proteins. For example, the identification of the ADAM22-binding domain of LGI1 as the target epitope of LGI1 antibodies led to the demonstration that the inhibition of LGI1-ADAM22 interaction is likely one of the main pathophysiological mechanisms of these antibodies [15]. Our study indicates that the extracellular Ig-like domain 2 is the immunodominant region of IgLON5 antibodies. The recognition of the binding domain is dependent on the tertiary structure, as it is not recognized by antibodies using immunoblots of denaturated IgLON5 [1], but the glycosylation of the epitope is not required for antibody recognition. Similar results have been obtained in the epitope analysis of other antibodies with predominant IgG4 subclass such as antibodies against muscle-specific kinase (MuSK), neurofascin 155 or contactin 1 that also recognize Ig-like domains [16–18].

In the current study using flow cytometry and CBA, we confirm that all patients have IgG4 IgLON5 antibodies, but they also have IgG1 antibodies. This is important because the mechanism of action of these subclasses of antibodies appear to be different in this disorder, as has been shown for other diseases. Although IgG4 antibodies were classically considered related to anti-inflammatory responses and unable to activate autoimmunity, several neurological disorders have been shown to be associated with IgG4 antibodies [19]. These include myasthenia gravis with antibodies against MuSK, encephalitis associated with antibodies against LGI1 or contactin-associated protein-like 2 (CASPR2), and subgroups of patients with Guillain-Barré syndrome or chronic inflammatory demyelinating polyneuropathy (CIDP) with antibodies against neurofascin 155, contactin 1, or CASPR1 [17]. In all these disorders, as it is the case of the anti-IgLON5 syndrome, antibodies are not only IgG4 but also present IgG1 and less frequently IgG2 or IgG3 antibodies [19].

In primary cultures of neurons, patients’ IgG antibodies crosslinked IgLON5 resulting in an irreversible decrease of the cell surface density of this protein in a time-dependent manner without affecting the number of excitatory synapses. Interestingly, this is in contrast with the reported effects of IgG1 antibodies from patients with encephalitis associated with anti-NMDA receptor or AMPA receptor encephalitis [6, 8, 20]. In these disorders, the antibodies cause an important decrease of the density of the corresponding targets, which is reversible upon removing the antibodies from the media. Moreover, in these two disorders, the immune response is associated with treatable forms of encephalitis whereas the anti-IgLON5 syndrome is usually refractory to immunotherapy [21]. Our current data, derived from experiments focused on the effects of IgG1 IgLON5 antibodies, do not rule out that the IgG4 antibodies may also have a pathogenic effect by other mechanisms independent of antigen internalization. For example, in myasthenia gravis associated with MuSK antibodies, both IgG1 and IgG4 antibodies affect acetylcholine receptor cluster stability in cultures of myotubes through different mechanisms, but only IgG4 antibodies can reproduce the disease in experimental animal models. Whereas a frequent pathogenic mechanism of IgG1 antibodies is to induce internalization of the antigen, IgG4 antibodies more often interfere with the normal protein-protein interactions of the target antigen, usually involved in cell adhesion or stabilization of receptors. A better knowledge of the proteins that normally interact with IgLON5 will be important to test the potential pathogenic role of the IgG4 class of IgLON5 antibodies.

Considering that the anti-IgLON5 syndrome usually has a protracted clinical course and is diagnosed many months after symptom onset (in contrast to NMDAR or AMPAR encephalitis which have a rapid presentation in days or weeks), our findings suggest that an early and prompt intervention with immunotherapy may potentially stabilize or decrease the reduction of IgLON5. At this time, it is unclear whether an early intervention would change the neuropathological findings of the disease, characterized by a unique distribution of neuronal-specific deposit of tau predominantly involving hypothalamus and tegmentum of brainstem [1]. If reduction of cell surface IgLON5 is linked to these deposits of tau, early immunotherapy may improve the often fatal outcome of anti-IgLON5 syndrome.

## Conclusions

Our study shows that IgLON5 antibodies from all 15 patients recognize the Ig-like domain 2 as the immunogenic region and the reactivity is not dependent on glycosylation. IgLON5 antibodies are predominantly IgG4, but all sera showed a variable amount of IgG1 antibodies with a mean of 33 % of the total IgG reactive with IgLON5. The effect of IgLON5 antibodies on hippocampal neuron cultures indicates an irreversible antibody-mediated downregulation of surface IgLON5 associated with an antibody-induced internalization. These effects were mediated by the IgG1 antibody subclass and support the potential pathogenic role of IgLON5 antibodies in the anti-IgLON5 syndrome.

## Additional files

Additional file 1: Figure S1. Purity of IgG1 and IgG4 fractions assessed by immunofluorescence on neurons (A) and HEK293 transfected with IgLON5 (B). The anti-IgG1 and IgG4 antibodies (green) show that there is no cross-contamination between fractions. Scale bar = 10 μm. **Figure S2.** Schematic diagram of the cloning strategy. Mutated clones from the human IgLON5 clone SC317071 (accession number NM_001101372.1, Origene) as template were generated by directed mutagenesis. The inserts are cloned in pCMV6 entry plasmid. Clones with different combinations of the three Ig domains of IgLON5 were designed but always including Ig3 that contains the epitope of the commercial antibody (epitope marked with a red line). Dashed lines indicate the deleted region. **Figure S3.** Example of an average experiment of flow cytometry analysis of IgLON5 antibodies subclasses. The patients’ serum has specific anti-IgLON5 IgG1 and IgG4 antibodies and it is negative for IgG2 and IgG3 antibodies. Logarithmic *X*-axis, red fluorescence, corresponds to the transfected cells and *Y*-axis, green fluorescence to the human serum binding. The Q2 (quadrant 2) contains the double positive cells. The same serum is incubated also in untransfected cells (bottom panels) as a control and to calculate the increment of mean fluorescence intensity (ΔMFI)). To calculate a cut-off threshold, five negative controls (normal human serum) were used. An average example is shown. **Figure S4.** IgLON5 clusters are highly expressed and distributed by the somatodendritic compartment of hippocampal neurons. A) The immunofluorescence shows a typical distribution of IgLON5 along the hippocampal dendrite. B) The graph shows the analysis of co-localization between IgLON5 clusters and synaptic markers (PSD95 and synapsin-I). The majority of the IgLON5 clusters were extrasynaptic 96.17 %, (SD 3.8). PSD95 co-localized with IgLON5 only in the 3.6 % (SD 0.5 %) and with synapsin-I in the 4 % (SD 0.4 %) of the spots measured by Imaris software. **Figure S5.** Effects of treatment for 3 days with IgG, Fab fragments, and divalent Fab fragments on cell surface IgLON5 clusters. A) Immunofluorescence on hippocampal neurons treated with Fab fragments of patients’ IgG do not produce any effect compared with IgG control or with Fab fragments of control IgG (Ctrl Fab). A clear decrease IgLON5 clusters can be observed when the Fab fragments from patients’ IgG are co-incubated with an anti-Fab secondary antibody. Scale bar = 3 μm. B) Quantification of the effect observed by immunofluorescence on treated neurons shown in (A). The effect of decrease of IgLON5 clusters is crosslinking dependent (*****p* < 0.0001). (DOCX 7161 kb)

## Abbreviations

CASPR2: Contactin-associated protein-like 2
CBA: Cell-based assay
CIDP: Chronic inflammatory demyelinating polyneuropathy
CSF: Cerebrospinal fluid
GPI: Glycosylphosphatidylinositol
LGI1: Leucine-rich glioma inactivated 1 protein
MFI: Mean fluorescence intensity
MuSK: Muscle-specific kinase
PBS: Phosphate buffered saline
PFA: Paraformaldehyde
PSD95: Post-synaptic density 95
PVDF: Polyvinylidene difluoride
REM: Rapid eye movement
