# Supplementary material for: Cellular investigations with human antibodies associated with the anti-IgLON5 syndrome
Source: J Neuroinflammation. 2016 Sep 1;13(1):226. doi: 10.1186/s12974-016-0689-1 (PMC5007989; doi:10.1186/s12974-016-0689-1)
Supplement: Additional file 1: Figure S1. — Purity of IgG1 and IgG4 fractions assessed by immunofluorescence on neurons (A) and HEK293 transfected with IgLON5 (B). The anti-IgG1 and IgG4 antibodies (green) show that there is no cross-contamination between fractions. Scale bar = 10 μm. Figure S2. Schematic diagram of the cloning strategy. Mutated clones from the human IgLON5 clone SC317071 (accession number NM_001101372.1, Origene) as template were generated by directed mutagenesis. The inserts are cloned in pCMV6 entry plasmid. Clones with different combinations of the three Ig domains of IgLON5 were designed but always including Ig3 that contains the epitope of the commercial antibody (epitope marked with a red line). Dashed lines indicate the deleted region. Figure S3. Example of an average experiment of flow cytometry analysis of IgLON5 antibodies subclasses. The patients’ serum has specific anti-IgLON5 IgG1 and IgG4 antibodies and it is negative for IgG2 and IgG3 antibodies. Logarithmic X-axis, red fluorescence, corresponds to the transfected cells and Y-axis, green fluorescence to the human serum binding. The Q2 (quadrant 2) contains the double positive cells. The same serum is incubated also in untransfected cells (bottom panels) as a control and to calculate the increment of mean fluorescence intensity (ΔMFI)). To calculate a cut-off threshold, five negative controls (normal human serum) were used. An average example is shown. Figure S4. IgLON5 clusters are highly expressed and distributed by the somatodendritic compartment of hippocampal neurons. A) The immunofluorescence shows a typical distribution of IgLON5 along the hippocampal dendrite. B) The graph shows the analysis of co-localization between IgLON5 clusters and synaptic markers (PSD95 and synapsin-I). The majority of the IgLON5 clusters were extrasynaptic 96.17 %, (SD 3.8). PSD95 co-localized with IgLON5 only in the 3.6 % (SD 0.5 %) and with synapsin-I in the 4 % (SD 0.4 %) of the spots measured by Imaris software. Figure S5. Effects of treatm [file 12974_2016_689_MOESM1_ESM.docx]

**Supplemental Material**

**Figure 1S**

**Figure 1S:** Purity of IgG1 and IgG4 fractions assessed by immunofluorescence on neurons (A) and HEK293 transfected with IgLON5 (B). The anti-IgG1 and IgG4 antibodies (green) show that there is no cross-contamination between fractions. Scale bar=10μm





**Figure 2S**


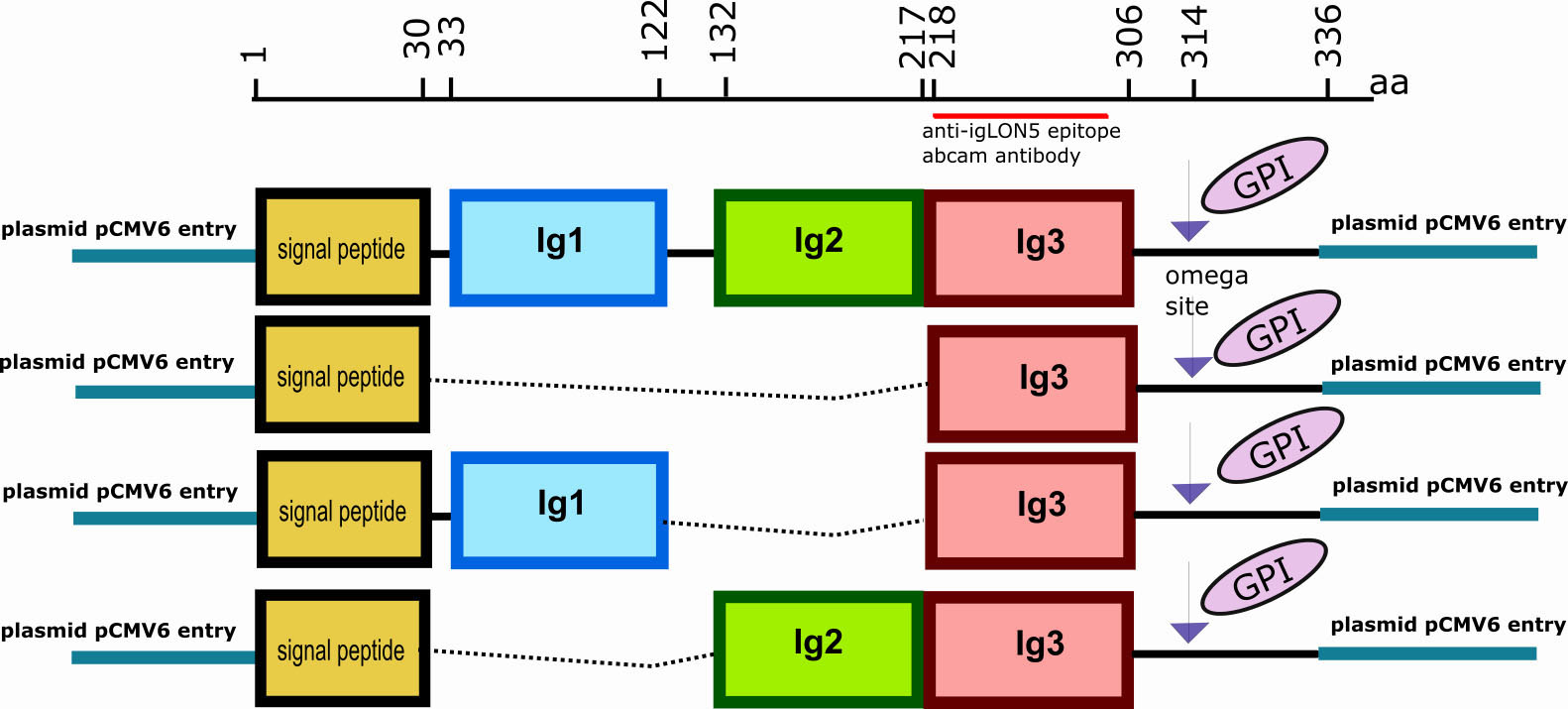


**Figure 2S:** Schematic diagram of the cloning strategy. Mutated clones from the human IgLON5 clone SC317071 (accession number NM_001101372.1, Origene) as template were generated by directed mutagenesis. The inserts are cloned in pCMV6 entry plasmid. Clones with different combinations of the three Ig domains of IgLON5 were designed but always including Ig3 that contains the epitope of the commercial antibody (epitope marked with a red line). Dashed lines indicate the deleted region.

**Figure 3S**


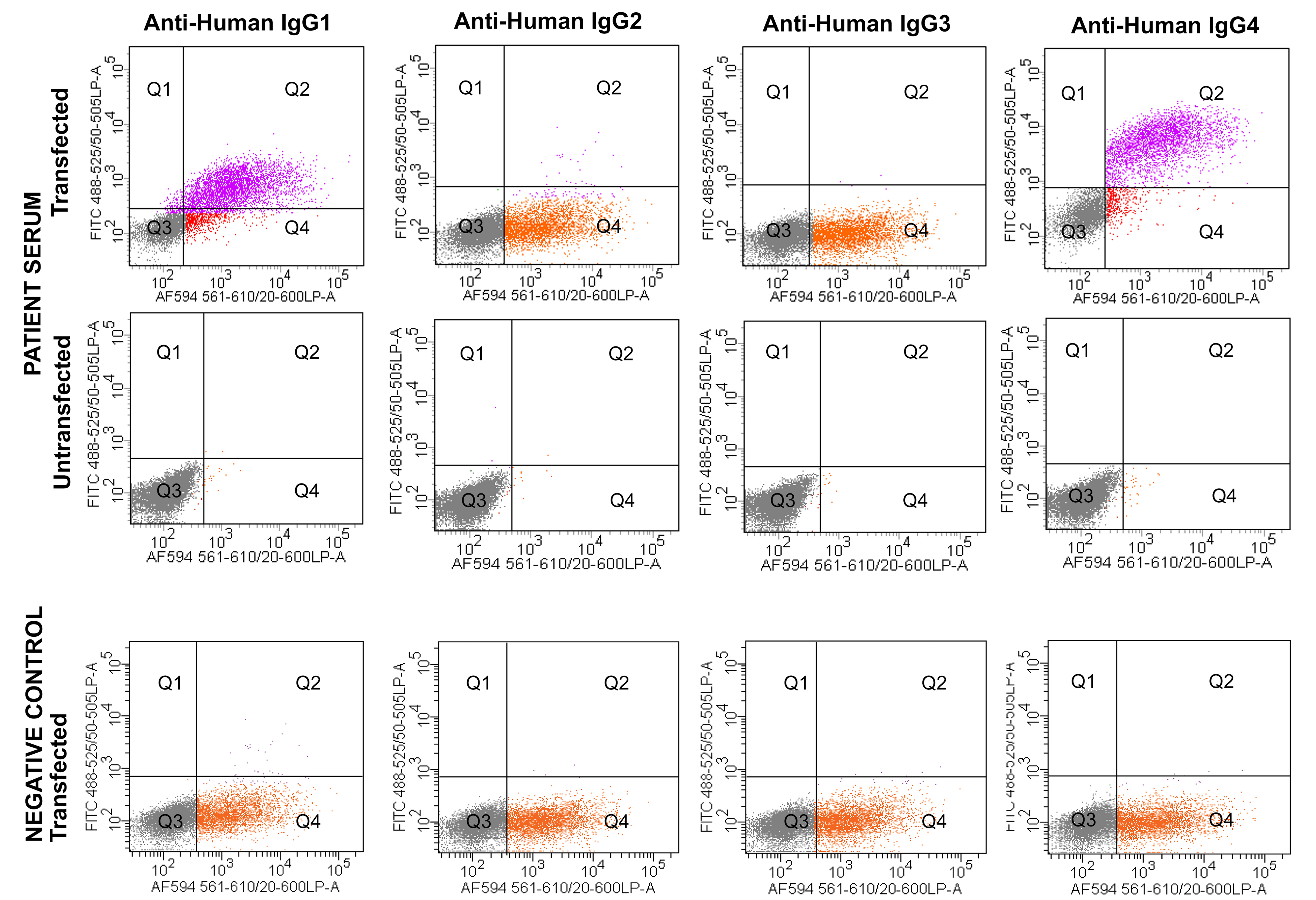


**Figure 3S:** Example of an average experiment of flow cytometry analysis of IgLON5 antibodies subclasses. The patient’s serum has specific anti-IgLON5 IgG1 and IgG4 antibodies and it is negative for IgG2 and IgG3 antibodies. Logarithmic X-axis, red fluorescence, corresponds to the transfected cells and Y-axis, green fluorescence to the human serum binding. The Q2 (quadrant 2) contains the double positive cells. The same serum is incubated also in untransfected cells (bottom panels) as a control and to calculate the increment of mean fluorescence intensity (ΔMFI). ). To calculate a cut-off threshold, 5 negative controls (normal human serum) were used. An average example is shown.

**Figure 4S**


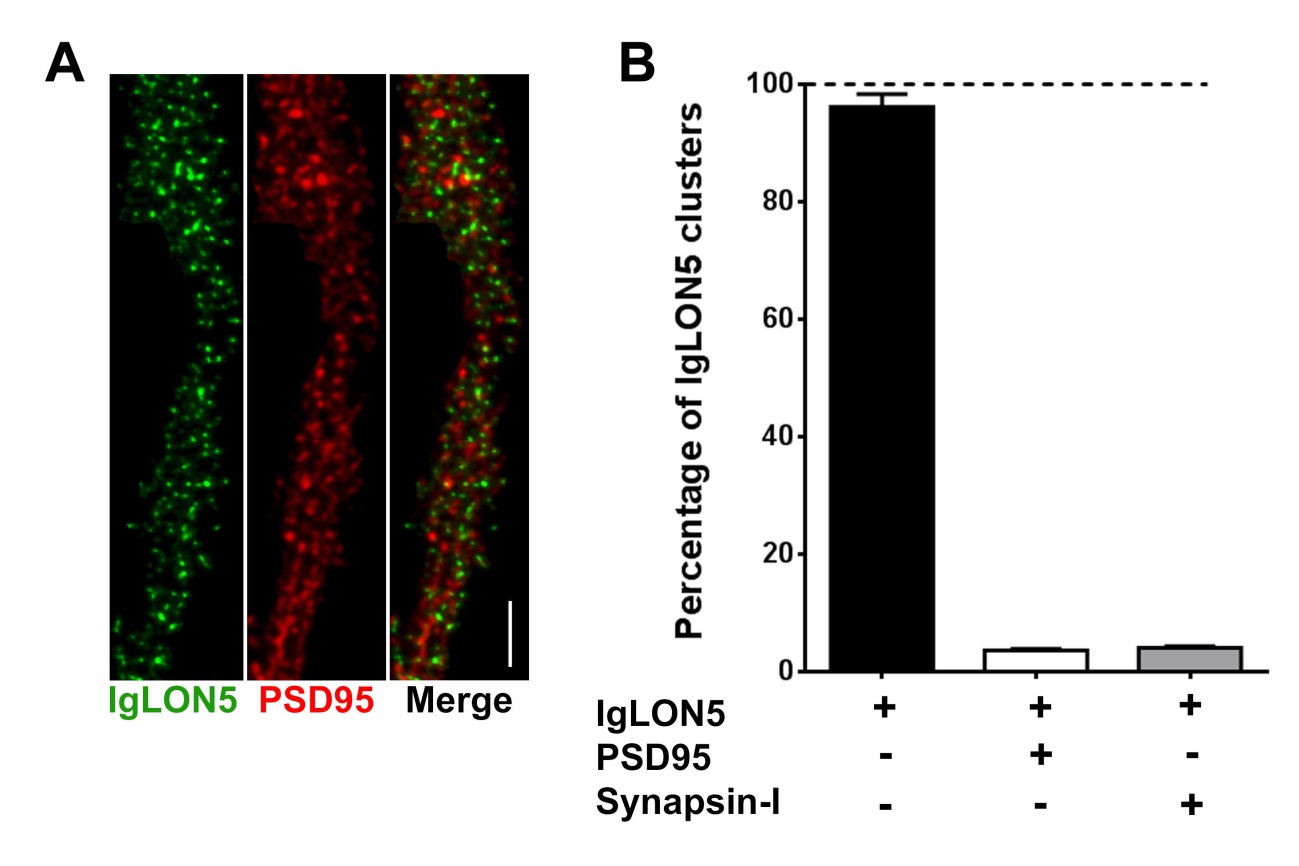


**Figure 4S:** IgLON5 clusters are highly expressed and distributed by the somatodendritic compartment of hippocampal neurons. A) The immunofluorescence shows a typical distribution of IgLON5 along the hippocampal dendrite. Scale bar=10 µm. B) The graph shows the analysis of colocalization between IgLON5 clusters and synaptic markers (PSD95 and synapsin I). The majority of the IgLON5 clusters were extrasynaptic 96.2% (SD 3.8). PSD95 colocalized with IgLON5 only in the 3.6% (SD 0.5%) and with Synapsin I in the 4% (SD 0.4%) of the spots measured by Imaris software.

**Figure 5S**


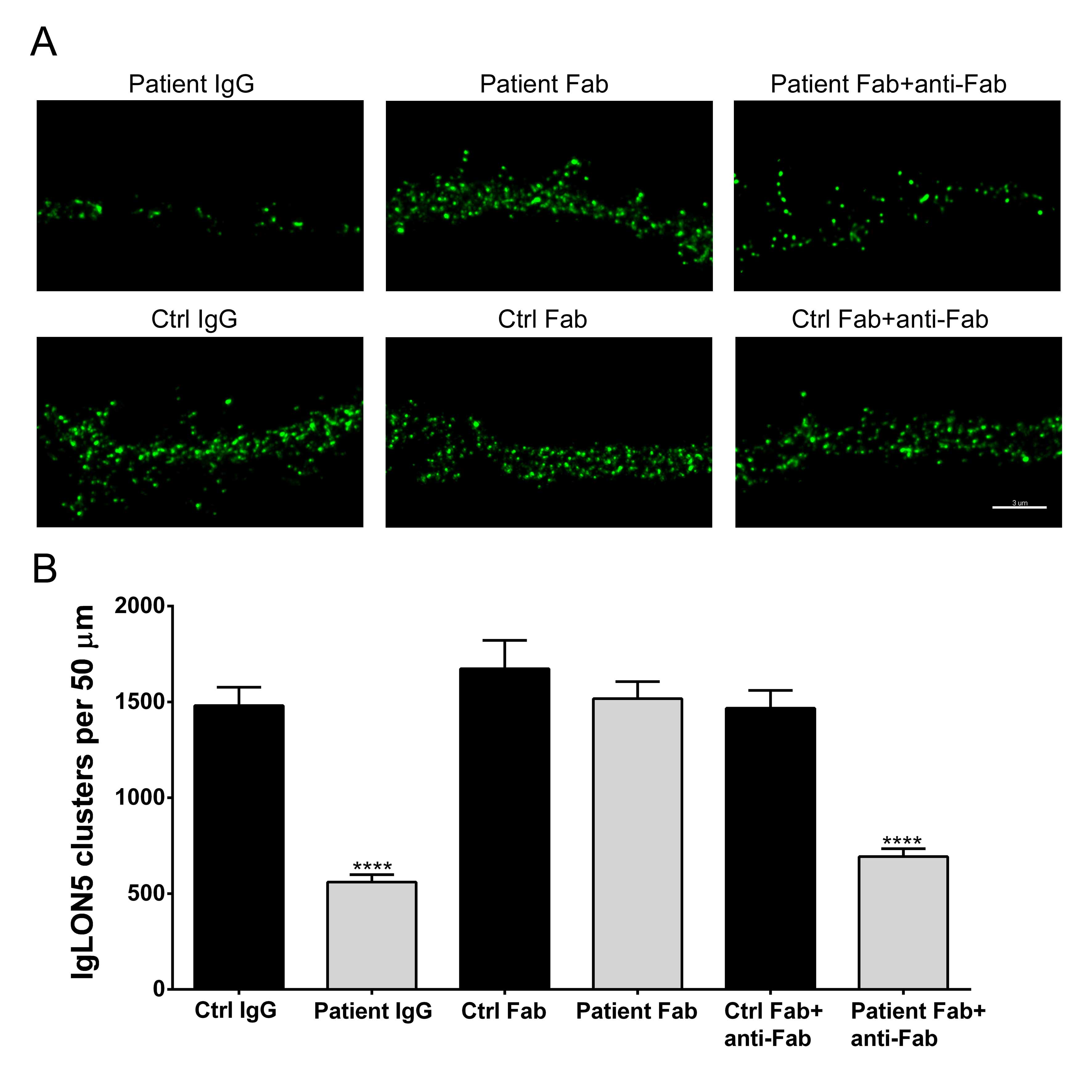


**Figure 5S.** Effects of treatment for 3 days with IgG, Fab fragments and divalent Fab fragments on cell surface IgLON5 clusters. A) Immunofluorescence on hippocampal neurons treated with Fab fragments of patient’s IgG do not produce any effect compared with IgG control or with Fab fragments of control IgG (Ctrl Fab). A clear decrease IgLON5 clusters can be observed when the Fab fragments from patient’s IgG are co-incubated with an anti-Fab secondary antibody. Scale bar=3 µm. B) Quantification of the effect observed by immunofluorescence on treated neurons shown in (A). The effect of decrease of IgLON5 clusters is crosslinking dependent (**** p<0.0001)
